# Income-related inequalities in chronic conditions, physical functioning and psychological distress among older people in Australia: cross-sectional findings from the 45 and up study

**DOI:** 10.1186/1471-2458-14-741

**Published:** 2014-07-22

**Authors:** Rosemary J Korda, Ellie Paige, Vasoontara Yiengprugsawan, Isabel Latz, Sharon Friel

**Affiliations:** 1 National Centre for Epidemiology and Population Health, Australian National University, Canberra ACT 0200, Australia; 2 School of Public Health, University of California, Los Angeles, USA

## Abstract

**Background:**

The burden of chronic disease continues to rise as populations age. There is relatively little published on the socioeconomic distribution of this burden in older people. This study quantifies absolute and relative income-related inequalities in prevalence of chronic diseases, severe physical functioning limitation and high psychological distress in mid-age and older people in Australia.

**Methods:**

Cross-sectional study of 208,450 participants in the 45 and Up Study, a population-based cohort of men and women aged 45–106 years from New South Wales, Australia. Chronic conditions included self-reported heart disease, diabetes, Parkinson’s disease, cancer and osteoarthritis; physical functioning limitation (severe/not) was measured using Medical Outcomes Study measures and psychological distress (high/not) using the Kessler Psychological Distress Scale. For each outcome, prevalence was estimated in relation to annual household income (6 categories). Prevalence differences (PDs) and ratios (PRs) were generated, comparing the lowest income category (<$20,000) to the highest (≥$70,000), using Poisson regression with robust standard errors, weighted for age, sex and region of residence. Analyses were stratified by age group (45–64, 65–79 and ≥80 years) and sex and adjusted for age and country of birth.

**Results:**

With few exceptions, there were income gradients in the prevalence of chronic conditions among all age-sex groups, with prevalence decreasing with increasing income. Of the chronic diseases, PDs were highest for diabetes (ranging between 5.69% and 10.36% across age-sex groups) and in women, also for osteoarthritis (5.72% to 8.14%); PRs were highest for osteoarthritis in men aged 45–64 years (4.01), otherwise they were highest for diabetes (1.78 to 3.43). Inequalities were very high for both physical functioning limitation and psychological distress, particularly among those aged 45–64 (PDs between 18.67% and 29.23% and PRs between 4.63 and 16.51). Absolute and relative inequalities tended to decrease with age, but remained relatively high for diabetes and physical functioning in the elderly (≥80 years).

**Conclusions:**

Significant inequalities in the prevalence of chronic conditions, physical functioning and psychological distress persist into old age. The additional health burden placed on those who are already disadvantaged is likely to become an increasingly important issue in an ageing population.

## Background

Chronic conditions account for the majority of the disease burden worldwide and this burden continues to rise, principally due to population ageing [1,2]. In Australia, chronic diseases make up around 80% of the total burden of disease [1,3]. As the proportion of people aged over the age of 65 years in this country is estimated to rise—from 13% to 23-25% by 2056, with the proportion of those 85 years and over rising from 1.6% to 4.9-7.3% [4]—the total chronic disease burden and associated functional limitations is expected to increase substantially.

A particular challenge of chronic disease in an ageing population is not just the total burden, but its socioeconomic distribution. It is well established that people who are socioeconomically disadvantaged are more likely to develop chronic disease than those who are more advantaged; they are also more likely to be economically vulnerable to the consequences. The economic difficulties arise not just through loss of income, which mainly affects working-age adults, but through direct costs associated with the illness, including out-of-pocket medical expenses and transport and carer costs, further compounding socioeconomic disadvantage [5-7]. Although there is some evidence that inequalities may diminish in older people, [8-11] even relatively small inequalities in the older population may increasingly become a health and social policy issue as the population ages, given the high prevalence of chronic disease and associated functional limitations in older people [3].

With rapid population ageing, knowledge of income-related inequalities in chronic disease and associated physical and mental health problems in later life will be important for designing appropriate health and welfare programs. Yet, while many studies on socioeconomic inequalities in the prevalence of chronic conditions include older people, data are usually aggregated for these older participants (often >65 years) e.g. [12-15], (with some exceptions, e.g. [9,16,17]), and there is very limited evidence of inequalities specifically among the oldest old (≥80 years) [9,18]. Further, relatively few studies present data on absolute inequalities, and in Australia, inequalities are often reported in relation to area-based measures of disadvantage, rather than at the individual or household level (e.g. [12]).

The aim of this paper is to quantify absolute and relative income-related inequalities in chronic diseases, physical functioning and psychological distress in mid-age and older Australians, using large-scale population-based study data that includes a large sample of people aged 80 years and over.

## Methods

### Participants

We used data from the 45 and Up Study, a cohort study involving over 250,000 men and women aged 45 and over from New South Wales (NSW), Australia; at the time of our study, we had data available on 266,848 participants. Participants in the Study were randomly sampled from the database of Medicare Australia, with over-sampling, by a factor of two, of individuals aged 80 years and over and people resident in rural areas. Around 10% of the entire NSW population aged 45 and over were included in the sample. Participants joined the Study by completing a baseline postal questionnaire, between January 2006 and March 2009. Further details of the Study are described in a separate publication [19], and questionnaires can be viewed at http://www.45andup.org.au.

### Study variables

#### Income

Participants were asked about their annual household income before tax from all sources, (including benefits, pensions and superannuation). Response options included eight income brackets, from “less than $5,000 per year” to “$70,000 or more per year”; the three lowest income brackets (less than $5,000, $5,000-$9,999 and $10,000-$19,999 per year) were combined for the analysis (see Table 1).

**Table 1:** Number and percentage of males and females in each age group, by annual household income category

	**Age group (years)**					
	**45-64**		**65-79**		**≥80**	
	**n**	**%**	**n**	**%**	**n**	**%**
**MALES**						
**<$20 K**	6,838	11.1	11,249	35.5	4,933	47.4
**$20- < 30 K**	4,116	6.7	6,095	19.2	2,113	20.3
**$30- < 40 k**	4,955	8.1	4,280	13.5	1,077	10.3
**$40- < 50 K**	5,891	9.6	3,041	9.6	714	6.9
**$50- < 70 K**	10,228	16.7	3,025	9.5	705	6.8
**≥** **$70 K**	29,272	47.8	4,040	12.7	876	8.4
**Total**	61,300	100.0	31,730	100.0	10,418	100.0
**FEMALES**						
**<$20 K**	11,962	16.5	12,275	50.0	5,108	64.4
**$20- < 30 K**	7,335	10.1	4,613	18.7	1,236	15.6
**$30- < 40 k**	7,438	10.3	2,734	11.1	567	7.1
**$40- < 50 K**	7,412	10.3	1,803	7.3	358	4.5
**$50- < 70 K**	11,764	16.3	1,674	6.8	355	4.5
**≥** **$70 K**	26,417	36.5	1,639	6.6	312	3.9
**Total**	72,328	100.0	24,738	100.0	7,936	100.0

#### Health outcomes

The 45 and Up survey includes variables on a large range of current and past health conditions. For this study we only examined variables that measured the presence of current chronic health conditions — heart disease, diabetes, Parkinson’s disease, cancer and osteoarthritis—and two outcomes commonly associated with chronic disease—physical functioning limitation and psychological distress [20-25]. Presence of heart disease, diabetes and Parkinson’s disease was determined by asking the participant to indicate if ‘a doctor ever told you that you have [that condition]’. Presence of osteoarthritis and cancer were determined by asking participants if ‘in the last month have you been treated for [that condition]’. Physical functioning was assessed using the Medical Outcomes Study physical functioning activity items, which are equivalent to items from the physical functioning scale of the SF-36 health survey [26]. The scale assesses functional capacity by inquiring about an individual’s ability to perform a range of moderate and vigorous physical tasks as well as everyday activities. The total MOS-PF score ranges from 0 to 100, and, as defined in previous studies [25,27], scores were categorised as severe physical functional limitation (<60) or not (≥60). Psychological distress was determined using the Kessler Psychological Distress Scale (K10) [28]. All items on the K10 begin with the phrase ‘during the past 4 weeks, about how often did you feel (…)’ followed by the description of an emotional state, such as: ‘tired out for no good reason?’ All answer options were based on a 5-point scale (‘none of the time’ (1), through to, ‘all of the time’ (5)). The K10 score has a range from 10 (no distress) to 50 (high distress) and, consistent with previous studies [25,29], scores of 22 and above were considered indicative of high levels of psychological distress.

### Statistical methods

We calculated the prevalence of chronic conditions in each income category, separately by age group (45–64, 65–79 and ≥80 years) and sex. We then used Poisson regression with robust variance estimation [30] to estimate prevalence differences (PDs, measures of absolute inequality) and prevalence ratios (PRs, measures of relative inequality), comparing prevalence in the lowest income category (<$20,000) to that in the highest (≥$70,000). Analyses were weighted for age, sex and region of residence (major city, rural, remote) using data from the 2006 Australian census [31] and adjusted for age (5-year age bands) and country of birth (categorised as Australia/New Zealand; Europe/North America; Asia; Africa/Middle East; other). Model fit was tested using residual plots and deviance and Pearson goodness-of-fit tests, which all confirmed appropriateness of fit of the Poisson model, in all analyses.

We also performed a sensitivity analysis to explore the magnitude and direction of any potential bias in the inequality estimates due to the exclusion of participants with missing income data. We did this by estimating PRs in the sample using area-level socioeconomic status (SES) as the SES measure (0.03% missing), comparing PRs in the full sample with those for whom income data were missing. Area-level SES was based on the Socio-Economic Indexes for Areas (SEIFA) Index of Relative Socioeconomic Disadvantage (IRSD) [32] derived from postcode of residence, and categorised into quintiles using cut-off scores from the 2006 Australian census. Analyses were carried out using Stata Version 13.1.

Ethics approval for this project was obtained from the NSW Population and Health Services Research Ethics Committee and the Australian National University Human Research Ethics Committee.

## Results

**Figure 1:**
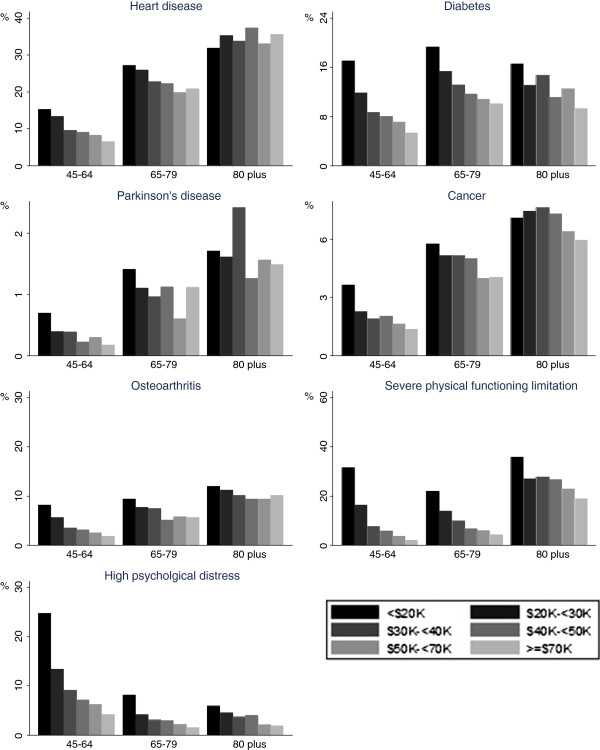
**Prevalence of chronic diseases, severe physical functioning limitation and high psychological distress by annual household income category, males.** Notes: 1. Different scale used for different outcomes. 2. Severe physical functioning limitation is a score <60 on the MOS-PF 3. High psychological distress is a Kessler Psychological Distress Scale score of ≥22.

After excluding participants with missing data on age (n = 752 (0.3%)) or household income (n = 57,646 (21.6%)), 208,450 participants (78%) were included in the analysis. Those most likely to have missing income data were female, older, of lower education and in poor health (p < .001 for all associations). The income profile of the final sample, by age and sex, is shown in Table 1. Household income varied by age, with the proportion of the high-income households decreasing, and low-income households increasing, with increasing age (p < .001 for both males and females).The prevalence of chronic conditions, severe physical functioning limitation and high psychological distress in each income category are shown separately by age group in Figures 1 (males) and 2 (females). Mostly there were clear socioeconomic gradients, with prevalence decreasing with increasing household income, the main exception being heart disease in men aged ≥80 years, where there was a reverse gradient.

**Figure 2:**
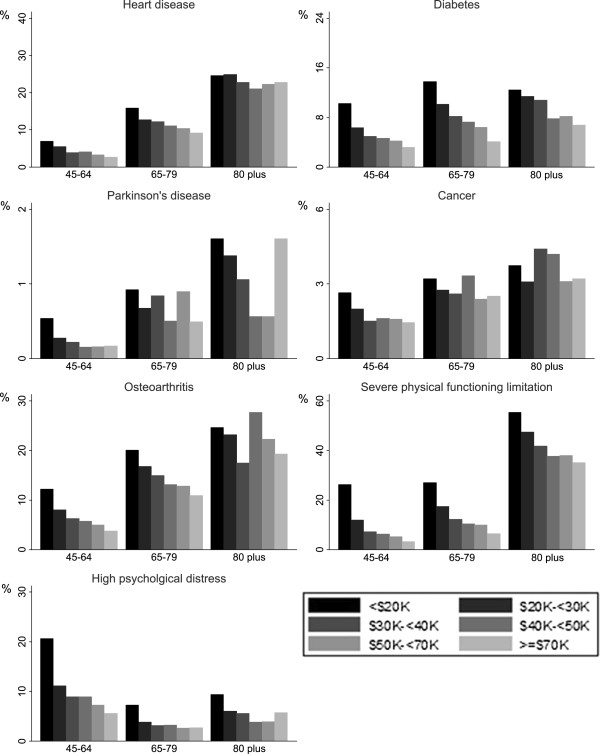
**Prevalence of chronic diseases, severe physical functioning limitation and high psychological distress by annual household income category, females.** Notes: 1. Different scale used for different outcomes. 2. Severe physical functioning limitation is a score <60 on the MOS-PF 3. High psychological distress is a Kessler Psychological Distress Scale score of ≥22.

Adjusted prevalence differences and prevalence ratios, comparing the lowest and highest household income groups, are shown in Tables 2 (males) and 3 (females). Although heart disease was the most prevalent chronic disease in males, absolute inequalities (PDs) were highest for diabetes, ranging from 7.16% (95% CI: 4.96-9.36%) in those aged ≥80 years to 10.36% (9.33-11.38%) in those aged 45–64 years. Relative inequalities (PRs) were also highest for diabetes in males aged 65–79 (PR = 1.98 (1.77-2.21)) and ≥80 years (PR = 1.78 (1.43-2.21)), but in, males aged 45–64 years, relative inequality was highest for osteoarthritis (PR = 4.01 (3.50-4.50)). For females, osteoarthritis was the most prevalent chronic disease (along with heart disease in those aged ≥80 years), with PDs also highest for this disease and for diabetes (with PDs of between 6 and 10% across all age groups for both diseases). Relative inequalities for females were also highest for diabetes in all age groups, ranging from 1.86 (1.21-2.84) in women aged over 80 years to 3.43 (2.62-4.48) in women aged 65–79. The PDs and PRs for physical functioning limitation and psychological distress were very high, particularly in those aged 45–64, with PDs of 29.23% (27.86-30.61%) in males and 22.91% (21.85-23.98%) in females for severe physical functioning limitation (respective PRs of 16.51 (14.90-18.29) and 8.15 (7.46-8.90)); and PDs of 24.19% (22.81-25.57%) in males and 18.67% (17.61-19.73%) in females for high psychological distress (respective PRs of 7.15 (6.61-7.73) and 4.63 (4.31-4.97)).

**Table 2:** Prevalence (%) of chronic conditions, severe physical functioning limitation and high psychological distress in the total group and the lowest and highest income groups, and prevalence differences (PD) and prevalence ratios (PR) (with 95% confidence intervals), amongst males, by age group

	**Age group (years)**		
	**45-64**	**65-79**	**≥80**
**Heart disease**			
Total	8.52 (8.28-8.76)	24.43 (23.91-24.96)	33.54 (32.63-34.45)
Low income	13.24 (12.39-14.08)	26.47 (25.56-27.38)	31.99 (30.67-33.31)
High income	7.14 (6.80-7.48)	22.58 (21.13-24.03)	35.00 (31.84-38.16)
PD	6.10 (5.17-7.02)	3.89 (2.16-5.62)	-3.01 (-6.44-0.42)
PR	1.85 (1.71-2.01)	1.17 (1.09-1.26)	0.91 (0.83-1.01)
**Diabetes**			
Total	7.86 (7.63-8.10)	14.92 (14.48-15.35)	14.28 (13.61-14.96)
Low income	16.02 (15.06-16.98)	19.33 (18.50-20.16)	16.40 (15.36-17.44)
High income	5.66 (5.36-5.97)	9.77 (8.78-10.77)	9.24 (7.31-11.17)
PD	10.36 (9.33-11.38)	9.56 (8.25-10.87)	7.16 (4.96-9.36)
PR	2.83 (2.61-3.07)	1.98 (1.77-2.21)	1.78 (1.43-2.21)
**Cancer**			
Total	1.75 (1.64-1.86)	4.92 (4.66-5.18)	7.01 (6.52-7.51)
Low income	3.23 (2.77-3.68)	5.49 (5.02-5.96)	7.06 (6.33-7.78)
High income	1.45 (1.29-1.60)	4.08 (3.40-4.76)	5.78 (4.24-7.31)
PD	1.78 (1.29-2.26)	1.41 (0.58-2.24)	1.28 (0.42-2.98)
PR	2.23 (1.86-2.67)	1.35 (1.12-1.62)	1.22 (0.92-1.62)
**Osteoarthritis**			
Total	2.91 (2.76-3.05)	7.45 (7.13-7.77)	11.12 (10.51-11.73)
Low income	7.42 (6.73-8.12)	9.16 (8.56-9.76)	11.95 (11.04-12.87)
High income	1.85 (1.68-2.03)	5.84 (5.04-6.65)	10.24 (8.20-12.27)
PD	5.57 (4.85-6.29)	3.32 (2.30-4.34)	1.72 (0.52-3.96)
PR	4.01 (3.50-4.59)	1.57 (1.34-1.83)	1.17 (0.94-1.45)
**Parkinson’s disease**			
Total	0.28 (0.23-0.33)	1.19 (1.05-1.32)	1.70 (1.45-1.95)
Low income	0.60 (0.39-0.80)	1.41 (1.16-1.65)	1.72 (1.35-2.09)
High income	0.17 (0.12-0.23)	1.33 (0.91-1.74)	1.48 (0.68-2.28)
PD	0.43 (0.21-0.64)	0.08 (-0.40-0.57)	0.24 (-0.64-1.12)
PR	3.45 (2.14-5.55)	1.06 (0.74-1.52)	1.16 (0.65-2.08)
**Severe physical functioning limitation**			
Total	6.42 (6.22-6.62)	12.69 (12.27-13.11)	29.86 (28.88-30.83)
Low income	31.12 (29.76-32.48)	21.46 (20.52-22.4)	35.42 (33.88-36.95)
High income	1.89 (1.71-2.06)	4.36 (3.64-5.07)	18.89 (16.12-21.66)
PD	29.23 (27.86-30.61)	17.10 (15.91-18.30)	16.53 (13.35-19.70)
PR	16.51 (14.9-18.29)	4.93 (4.15-5.85)	1.87 (1.61-2.18)
**High psychological distress**			
Total	7.68 (7.45-7.91)	4.61 (4.34-4.88)	4.61 (4.15-5.08)
Low income	28.13 (26.77-29.48)	8.58 (7.92-9.24)	5.85 (5.07-6.63)
High income	3.94 (3.70-4.18)	1.36 (0.97-1.75)	1.92 (0.92-2.93)
PD	24.19 (22.81-25.57)	7.21 (6.44-7.98)	3.93 (2.66-5.20)
PR	7.15 (6.61-7.73)	6.29 (4.68-8.46)	3.04 (1.78-5.22)

Notes: 1. Severe physical functioning limitation is a score <60 on the MOS-PF 2. High psychological distress is a Kessler Psychological Distress Scale score of ≥22. 3. Analyses are weighted for age group, sex and region, and adjusted for age (5-year age groups) and country of birth.

**Table 3:** Prevalence (%) of chronic conditions, severe physical functioning limitation and high psychological distress in the total group and the lowest and highest income groups, and prevalence differences (PD) and prevalence ratios (PR) (with 95% confidence intervals), amongst females, by age group

	**Age group (years)**		
	**45-64**	**65-79**	**≥80**
**Heart disease**			
Total	3.83 (3.68-3.99)	13.69 (13.20-14.18)	24.30 (23.35-25.26)
Low income	5.85 (5.38-6.32)	15.42 (14.69-16.15)	24.77 (23.56-25.97)
High income	2.95 (2.70-3.19)	9.74 (8.09-11.40)	22.90 (18.17-27.63)
PD	2.90 (2.37-3.44)	5.67 (3.86-7.49)	1.87 (-3.01-6.75)
PR	1.99 (1.76-2.23)	1.58 (1.33-1.89)	1.08 (0.87-1.34)
**Diabetes**			
Total	5.15 (4.97-5.34)	10.78 (10.34-11.22)	11.44 (10.73-12.14)
Low income	9.68 (9.07-10.29)	13.58 (12.88-14.27)	12.33 (11.42-13.24)
High income	3.50 (3.24-3.77)	3.96 (2.92-5.01)	6.64 (3.86-9.41)
PD	6.18 (5.50-6.85)	9.61 (8.35-10.87)	5.69 (2.77-8.62)
PR	2.76 (2.50-3.05)	3.43 (2.62-4.48)	1.86 (1.21-2.84)
**Cancer**			
Total	1.70 (1.59-1.81)	2.98 (2.73-3.22)	3.72 (3.30-4.15)
Low income	2.50 (2.18-2.83)	3.23 (2.86-3.59)	3.85 (3.31-4.39)
High income	1.54 (1.37-1.71)	2.57 (1.71-3.42)	2.97 (1.13-4.81)
PD	0.96 (0.59-1.34)	0.66 (0.27-1.60)	0.88 (-1.04-2.80)
PR	1.63 (1.36-1.94)	1.26 (0.88-1.79)	1.30 (0.69-2.45)
**Osteoarthritis**			
Total	6.07 (5.88-6.27)	17.29 (16.75-17.83)	23.78 (22.83-24.73)
Low income	10.82 (10.20-11.44)	19.90 (19.09-20.72)	24.76 (23.55-25.96)
High income	4.23 (3.94-4.52)	11.76 (9.97-13.55)	19.04 (14.65-23.42)
PD	6.59 (5.89-7.29)	8.14 (6.17-10.12)	5.72 (1.17-10.27)
PR	2.56 (2.33-2.81)	1.69 (1.44-1.98)	1.30 (1.03-1.65)
**Parkinson’s disease**			
Total	0.22 (0.19-0.26)	0.86 (0.73-1.00)	1.42 (1.16-1.68)
Low income	0.48 (0.35-0.62)	1.00 (0.79-1.21)	1.57 (1.23-1.91)
High income	0.17 (0.11-0.22)	0.36 (0.07-0.66)	1.58 (0.18-2.98)
PD	0.32 (0.16-0.47)	0.64 (0.28-1.00)	0.01 (-1.45-1.43)
PR	2.88 (1.84-4.50)	2.75 (1.19-6.35)	0.99 (0.40-2.46)
**Severe physical functioning limitation**			
Total	8.28 (8.05-8.51)	19.47 (18.87-20.07)	50.59 (49.35-51.83)
Low income	26.12 (25.09-27.14)	26.52 (25.52-27.52)	54.84 (53.27-56.42)
High income	3.20 (2.96-3.45)	7.50 (5.93-9.06)	36.53 (30.51-42.55)
PD	22.91 (21.85-23.98)	19.02 (17.15-20.89)	18.31 (12.08-24.55)
PR	8.15 (7.46-8.90)	3.54 (2.86-4.37)	1.50 (1.27-1.78)
**High psychological distress**			
Total	9.35 (9.11-9.59)	5.30 (4.95-5.66)	7.99 (7.27-8.71)
Low income	23.82 (22.81-24.83)	7.64 (7.02-8.26)	9.18 (8.23-10.13)
High income	5.15 (4.85-5.44)	2.73 (1.81-3.65)	6.56 (3.24-9.87)
PD	18.67 (17.61-19.73)	4.91 (3.80-6.02)	2.62 (0.83-6.07)
PR	4.63 (4.31-4.97)	2.80 (1.98-3.96)	1.40 (0.84-2.35)

Notes: 1. Severe physical functioning limitation is a score <60 on the MOS-PF 2. High psychological distress is a Kessler Psychological Distress Scale score of ≥22. 3. Analyses are weighted for age group, sex and region, and adjusted for age (5-year age groups) and country of birth.

Broad comparisons of inequalities across age groups show that, although prevalence of chronic disease and severe physical functioning limitation (but not high psychological distress) increased with age, in men both absolute (PDs) and relative inequalities (PRs) were highest in the youngest age group (45–64); in women absolute inequalities were highest in the two youngest age groups (45–64 and 65–79), and relative inequalities (PRs) were, with the exception of diabetes, always highest in the youngest age group (45–64).

The sensitivity analysis showed there was no change in the direction, and little difference in magnitude, of the PRs based on area-disadvantage among the sample with income missing compared to the full sample (results available on request).

## Discussion

Substantial socioeconomic inequalities in chronic disease, physical functioning and psychological distress are evident in mid-age and older adults in Australia, with the prevalence of these conditions generally increasing with decreasing household income. Our findings provide empirical evidence of income-related inequalities not just in working-age individuals, but also older people, including the oldest old, where the overall burden of chronic disease is very high. The PRs reported in this study are greater than those seen for many other chronic disease risk factors, bearing in mind socioeconomic inequalities are, to some extent, likely to reflect the inequalities in prevalence of these other risk factors (e.g. smoking) [33]. The particularly high levels of diabetes, osteoarthritis, physical functioning limitations and psychological distress amongst people in lower income households is especially concerning, both from an individual and population perspective, given the high associated health care needs and costs [34].

Our results are generally consistent with those of other Australian and international studies of mid-age and older age people (>65 years), which report significant socioeconomic inequalities in the prevalence of chronic diseases, physical functional limitations and mental health problems, across different measures of SES, although not necessarily for all health conditions [7-10,12-18,35-41]; an earlier Australian report actually showed reverse gradients in cancer [13], a pattern also reported in older-age Europeans (aged 60–79 years) [16]. Our study adds to the limited empirical evidence, conducted in the 1990s, on inequalities in the oldest old (≥80 years). A cross-sectional study involving 11 European countries found income and educational inequalities in reductions in daily activities due to physical or mental health problems, and long-term disabilities, in the 60–69 and 70–79 year age groups but, unlike our study, not in those aged ≥80 years (except for disabilities in men ≥80 years) [9]. In a Swedish study, clear occupational class inequalities in self-reported circulatory problems and “aches and pains”, as well as measured lung function, were reported in people aged 77–98 years [18].

Of the chronic diseases examined in our study, absolute and relative inequalities were highest for diabetes and osteoarthritis. This contrasts to an earlier report of income-related relative inequalities in chronic disease in Australia (2001 data), where gradients in these diseases were not always evident (only for diabetes in females aged 25–64 and males aged ≥65, and for arthritis in those aged 25–64 (absolute inequalities not reported)) [13]. However, our findings are similar to those from a large study of pooled national surveys (1990s) involving eight European countries, which also found relative inequalities were high for diabetes and arthritis (as well as stroke and diseases of the nervous system) [16]. More recent international studies (2004–2008) show modest absolute and relative income inequalities in diabetes in some countries, but not in others [14,17,40]. While difficult to make direct comparisons between studies, differences in findings are likely to reflect difference across time and place in underlying social conditions and risk factors, such as obesity, physical inactivity and smoking. With regard to the finding of large inequalities in physical functional limitations, this has been consistently reported among mid age and older people in previous studies [9,15,17,35-37,39,41]. Our finding of substantial inequalities in high psychological distress, particularly among younger people, is also consistent with the weight of evidence, showing higher prevalence of mental health problems among lower socioeconomic groups [42], although inequalities in older people have been less consistently reported [17,35,43].

While we did not directly test if the extent of inequality in chronic conditions differed significantly across age groups, we found absolute and relative inequalities were for the most part smaller in the older adults. This is broadly consistent with the findings from other cross-sectional studies, showing relative, but not necessarily absolute, inequalities tend to diminish with age [9,16]; and with longitudinal studies in Australia [10] and the U.S. [8], showing widening of absolute inequalities in physical impairment in mid-adult years, but narrowing in older ages. Nevertheless, at a population level, with the combination of high overall prevalence of chronic disease and high proportion of low-income households in the very old, the total excess burden associated with low income is likely to be substantial in this age group.

It was not the aim of this paper to determine causality between income and chronic disease, which is not possible with these cross-sectional data and with limited adjustment of covariates. Rather, by quantifying the extent of inequality in chronic conditions in different age groups we were able to show that the burden of disease is greater among individuals in lower income households, a finding which holds true even into older age, albeit to a relatively smaller extent. These findings are likely to reflect several factors, including inequalities in incidence (albeit not necessarily at older ages [44]), arising through multiple mechanisms [45]. Moreover, it is likely that the income gradients in these conditions to some extent reflect the known socioeconomic gradients in risk factors for chronic disease, including smoking, obesity and physical inactivity [13,34], as well as other inequalities, such as use of health care e.g. [46-48]. The findings also reflect survival, as well as ‘reverse causality’. In particular, the very high income-related inequalities seen in physical function in the 45–64 age-group in this study are likely to be explained at least in part by the fact that while socioeconomic disadvantage is a risk factor for poor health, poor health also affects income, particularly in people of working age [49]. The lower relative inequalities in older people may partly reflect the ‘survivor effect’, whereby the negative effect of low SES on health means those remaining in the cohort are not a random sample of the population but rather reflect those who are more likely to have survived the effect of low SES on premature mortality [11].

Strengths of this study include that it is an order of magnitude larger than previous national and international studies investigating the relationship of SES to health outcomes in mid-age and older people; and the over-sampling of individuals aged over 80 years meant that we had the power to examine variations in outcomes in the very old, this increasingly important group as the population ages, yet one which is often excluded (or at best aggregated) in population health studies.

Limitations of this study include that it is based on self-reported health outcomes, and that the physical functional limitations and psychological distress measures may not necessarily reflect chronic conditions. Further, like many population-based cohort studies, the response rate was low (~18%). It was decided at study conception that the main concern was to obtain a large sample, with data across a wide variety of exposures rather than focusing on response rates. As such, while the sample was randomly drawn, it is unlikely to be representative of the general population and thus caution should be used when interpreting and generalising absolute prevalence estimates. However, representativeness is not necessary for reliable estimates of relative risks based on internal comparisons within study populations, and it has been shown that the relative measures estimated from non-representative studies are consistent with the relative measures estimated from representative studies [50,51]. As such, the relative inequality estimates, as measured in this study, are assumed to be valid and broadly generalisable, particularly if it can be assumed that the extent of errors in self-reporting do not differ by income or by selection into the study. Similarly, although inequality estimates may be biased due to the exclusion of those with missing income data, this is only likely to be the case if the strength of association between SES and health is different in those excluded, and the results of the sensitivity analysis suggested this was not the case.

## Conclusion

There has been some uncertainty as to the magnitude of health inequality in older ages, and indeed whether the magnitude of inequality is meaningful. This study suggests it is, with significant inequalities in the prevalence of chronic conditions, severe physical limitations and high psychological distress evident among elderly people. The increased burden in these groups highlights the importance of considering the wider implications of inequalities in chronic conditions among these age groups, including the costs borne by individuals and households in managing chronic illness, and how this places further burden on those in the community who are already disadvantaged. The findings also pose the policy challenge of needing to prevent such inequalities in older years, which arguably manifest much earlier in peoples’ lives. These health inequalities are likely to become an increasingly important economic and social issue in an ageing population.

## Competing interests

The authors declare that they have no competing interests.

## Authors’ contributions

RK designed and coordinated the study, and drafted the manuscript. IL and EP carried out the statistical analysis and helped draft the manuscript. VY and SF participated in the design of the study and helped draft the manuscript. All authors read and approved the final manuscript.

## Pre-publication history

The pre-publication history for this paper can be accessed here:

http://www.biomedcentral.com/1471-2458/14/741/prepub
